# A cocktail vaccine with monkeypox virus antigens confers protection without selecting mutations in potential immune evasion genes in the vaccinia WR strain challenge

**DOI:** 10.1128/mbio.03200-25

**Published:** 2025-12-31

**Authors:** Xintong Sun, Luhua Zhang, Guohua Chen, Fan Yang, Xiaoyu Ning, Jinxin Qiu, Yuxuan Gao, Jianshe Yang, Wenhui Zhang, Zihui Zhang, Yueyue Zhang, Siyuan Li, Mingcong Zeng, Baoquan Fu, Yongfeng Li, Chen Peng, Weike Li

**Affiliations:** 1 State Key Laboratory of Animal Disease Control and Prevention, Lanzhou Veterinary Research Institute, Chinese Academy of Agricultural Sciences 555071 , Lanzhou, China; 2 Gansu Province Research Center for Basic Disciplines of Pathogen Biology 555174 , Lanzhou, China; 3 Division of Respiratory and Critical Care Medicine, State Key Laboratory of Biotherapy, West China Hospital of Sichuan University and Collaborative Innovation Center of Biotherapy 34753, Chengdu, China; 4 State Key Laboratory of Animal Disease Control and Prevention, Harbin Veterinary Research Institute, Chinese Academy of Agricultural Sciences 687216 , Harbin, China; 5 National Key Laboratory of Veterinary Public Health and Safety, Key Laboratory of Animal Epidemiology of the Ministry of Agriculture and Rural Affairs, College of Veterinary Medicine, China Agricultural University 34752, Beijing, China; 6 School of Life Sciences, Lanzhou University 12426, Lanzhou, China; Huazhong Agricultural University, Wuhan, Hubei, China

**Keywords:** monkeypox virus, cocktail vaccine, viral evolution, vesicular stomatitis virus vector, cross-protective immunity

## Abstract

Faced with the global monkeypox outbreak, current vaccine development predominantly focuses on the mRNA platform despite its limitations in stability and long-term efficacy. Here, we engineered a recombinant vesicular stomatitis virus (rVSV)-vectored cocktail vaccine encoding four conserved monkeypox virus (MPXV) antigens (A35R, A29L, M1R, and B6R; >94% clade homology), leveraging the thermostable properties of the VSV platform validated for 4°C storage in Ebola vaccines. In BALB/c mice, this multi-antigen vaccine elicited a rapid humoral response with specific IgG detectable by day 7, effectively neutralized the virus, and induced a robust Th1/Th2 balanced cytokine response. Immunization conferred 100% survival against lethal vaccinia virus WR strain challenge, with undetectable viral loads in the lungs and serum, and sustained efficacy against secondary infection at 60 days. Histopathology confirmed minimal lung damage in vaccinated mice. Crucially, upon the successive challenges, mutations in key poxvirus immune evasion genes (E3L and B7R) emerged in the single-component vaccine groups but were absent in the cocktail vaccine group. This finding provides direct evidence that the cocktail strategy suppresses viral escape, underscoring a fundamental advantage over single-antigen approaches. Our findings demonstrate the rVSV-based cocktail vaccines as a potent, scalable, and thermostable candidate for global MPXV control, particularly in regions with limited settings.

**IMPORTANCE:**

The global emergence of the monkeypox virus (MPXV) underscores the urgent need for effective and accessible vaccines. We developed a recombinant vesicular stomatitis virus (rVSV)-vectored cocktail vaccine expressing four conserved MPXV antigens. This multivalent vaccine elicits rapid and potent immune responses in mice, conferring complete protection against lethal vaccinia virus challenge. A critical finding is that while successive viral challenges selected for mutations in key immune evasion proteins in single-antigen vaccine groups, these mutations were absent in the cocktail-vaccinated group. This suggests that the cocktail strategy may suppress viral genetic drift, potentially limiting escape pathways. Combined with the thermostability of the VSV platform, our vaccine presents a promising and scalable candidate for combating monkeypox.

## INTRODUCTION

The infectious disease known as monkeypox virus (MPXV) can cause a range of symptoms, including a painful rash, swollen lymph nodes, fever, headache, back pain, muscular aches, and flu-like symptoms (1). On 14 August 2024, due to a new variant and its broader global spread, the monkeypox epidemic was recently declared a public health emergency of international concern by the World Health Organization. Since the alerts on 23 July 2022 and 11 May 2023, this is the third-highest level MPXV alert, implying prophylactic and therapeutic measures are an urgent need.

Because of the highly homologous genomes and strong cross-protection between MPXV and smallpox, three pox vaccines have been licensed for use against MPXV: ACAM2000, a live attenuated smallpox vaccine (2, 3); JYNNEOS, a replication-deficient smallpox vaccine (4, 5); and APSV, a replication-competent vaccinia virus (VACV) (2, 6–10). However, safety concerns remain. For example, ACAM2000 may lead to adverse effects, such as rash, fever, and myalgia, and complications from the VACV can be severe in immunocompromised individuals. JYNNEOS and APSV are available for smallpox emergencies and therefore have only limited accessibility. Encouragingly, emerging data on next-generation mRNA vaccines suggest that MPXV mRNA vaccinations protect against lethal VACV and MPXV challenges (9, 11–14). However, further long-term studies are essential to determine the durability of the immune response and to address potential allergic reactions in some individuals (15). Moreover, the logistical challenges associated with mRNA vaccine deployment, particularly their stringent cold-chain requirements (e.g., storage at −70°C for BNT162b2), limit accessibility in resource-limited regions where MPXV remains endemic (15, 16).

Recombinant viral vectors, such as the vesicular stomatitis virus (VSV), have long been used to deliver specific disease antigens (17). The VSV platform has several advantages as a vaccine vector, including its non-pathogenic replication in various cell types and its ability to elicit robust antibody and cell-mediated immune responses against foreign antigens (18–20). Compared to other types of vaccines, such as mRNA and inactivated vaccines, recombinant vector vaccines exhibit superior thermal stability (21). The thermostability of VSV-based vaccines is attributed to the intrinsic structural resilience of the viral envelope and nucleocapsid, which maintain integrity under fluctuating temperatures (22, 23). Genetic engineering approaches, such as codon optimization of antigen inserts and stabilization of the viral glycoprotein, further enhance thermal tolerance, enabling long-term storage at 4°C without significant loss of infectivity (24–26). Additionally, the thermal stability of recombinant vector vaccines can be significantly enhanced through genetic engineering modifications or formulation optimization due to the unique viral particle structure, thereby further reducing storage and transportation costs (24, 27, 28). Despite its relatively small RNA genome, VSV can accommodate large foreign genes and ensure natural protein folding. In addition, VSV has an extremely low seroprevalence in the general population (29, 30). VSV does not undergo recombination and replicates exclusively through RNA intermediates in the cytoplasm. Recombinant VSV (rVSV) generated using reverse genetics is less pathogenic (attenuated) than wild-type strains (30, 31). The clinical success of the VSV-vectored Ebola vaccine (ERVEBO) underscores the translational potential of this platform. The rVSV-ΔG-ZEBOV-GP vaccine, approved by the FDA (32), demonstrated safety in humans and efficacy against the Ebola virus (EBOV) in a Phase III clinical trial, underscoring the potential of VSV as a vaccine vector (33–36).

Cocktail vaccines induce combined immunity by incorporating multiple antigens or various pathogen strains and have long been used to prevent infections caused by complex or highly variable pathogens, eliciting broader neutralizing antibody and T-cell responses. Classic examples include the application against influenza virus, African swine fever virus, and hepatitis B virus infections (37–39). Interestingly, under the vaccine-induced pressure, viruses may acquire immune escape either through amino acid substitutions within critical epitope regions or by modulating the sequence and expression of certain immune regulatory genes. This suggests that single-component vaccines are more prone to partial escape, whereas cocktail vaccines, by targeting multiple structural proteins and immune pathways, can markedly increase the evolutionary cost of viral escape. Therefore, investigating whether single-component or mixed immunization alters the structure or function of these key proteins is of great significance for understanding how mixed vaccines suppress viral immune evasion.

In the present study, we engineered recombinant VSV-based immunization cocktails expressing the A35R, A29L, M1R, and B6R proteins of MPXV, which are recognized as specific antigenic targets (11). The vaccines were evaluated in a BALB/c mouse model, with robust, high-titer humoral and cellular immune responses against rVSV-based cocktail vaccinations. The immunized mice exhibited complete protection when challenged with a lethal dose of the VACV-WR strain, and as a secondary challenge in mice that had previously recovered from VACV. Notably, our studies demonstrated that repeated non-lethal viral challenge induced amino acid mutations in immune-evasion-associated proteins (E3L and B7R) in mice immunized with single-component vaccines, whereas the cocktail vaccine completely prevented the occurrence of these mutations. Our findings not only contribute to the development of a diverse vaccine platform but also provide direct evidence that cocktail strategies confer superior protection by suppressing viral escape.

## RESULTS

### Recovery of recombinant VSVs expressing MPXV antigens

**Fig 1:**
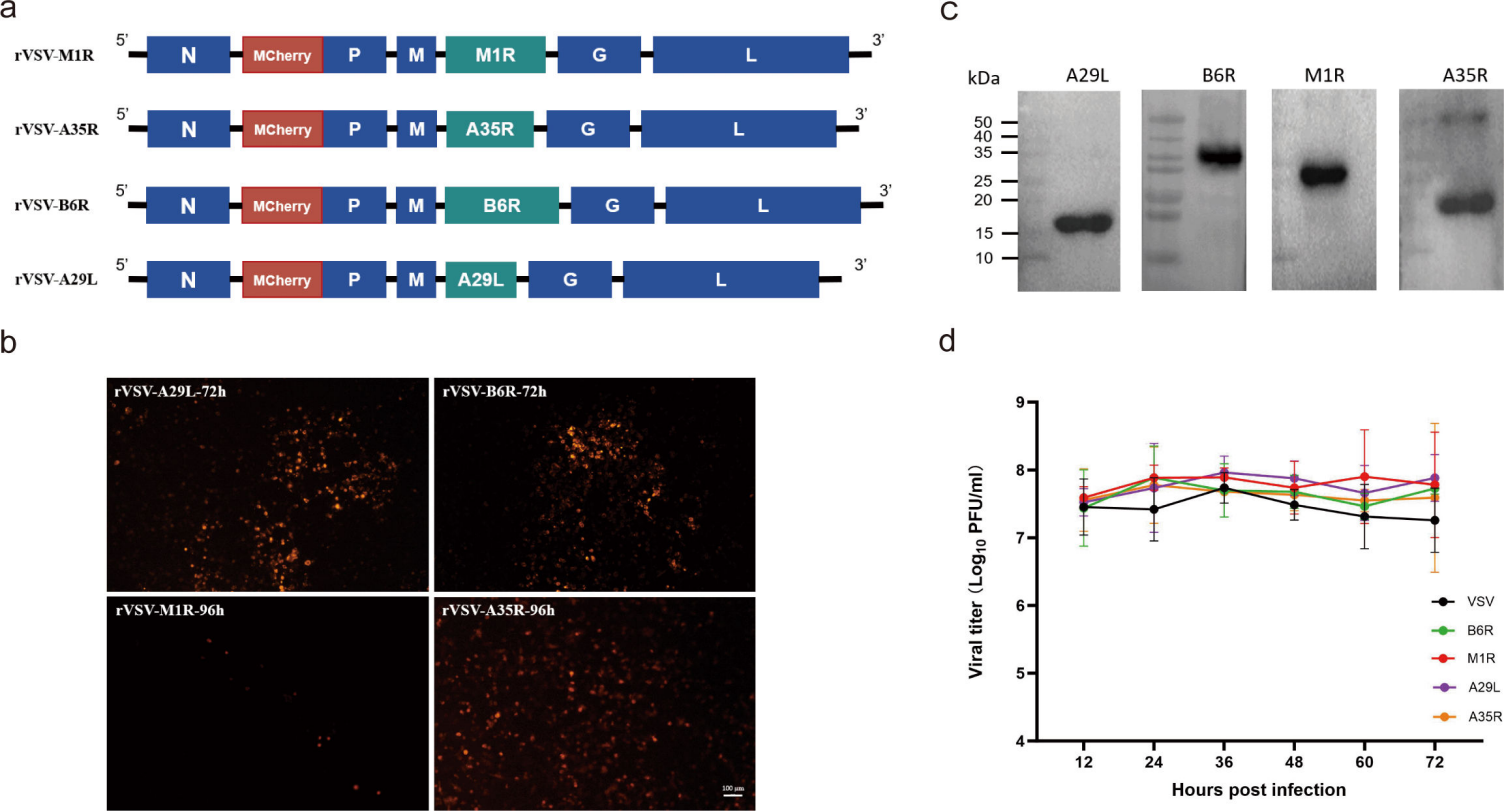
Preparation and validation of rVSV-expressing MPXV antigens. (**a**) Design of rVSVs. The codon sequences of MPXV *A35R* (546 bp), *A29L* (397 bp), *B6R* (986 bp), and *M1R* (753 bp) were inserted into the VSV backbone between the M and G proteins. Additionally, an mCherry protein was fused at the N-terminus of the P protein. (**b**) Fluorescence signal of the rescued rVSVs. Viruses obtained through reverse genetics, with images captured 72–96 h after suspension transfection. Five fields of view were analyzed for each virus. (**c**) Expression of MPXV antigen proteins by the VSV vector. BHK-21 cells were infected with each rVSV at an MOI of 1.0. At 24 h post-infection, cells were lysed in 500 μL of lysis buffer. Subsequently, 10 μL of the lysate was analyzed by Western blot using anti-MPXV A35R, A29L, B6R, and M1R monoclonal antibodies to confirm the expression of the MPXV antigens; the sizes of each protein were A35R −20.0 kDa, A29L −14.5 kDa, B6R −36.2 kDa, M1R-27.6 kDa, respectively. (**d**) One-step growth curve validation of the rVSVs. BHK-21 cells were infected with each recombinant virus at an MOI of 0.01. The virus was collected at various time points (0, 12, 24, 36, 48, 60, and 72 h), and the virus titer was measured by PFU assay. Each sample was tested in triplicate.

To engineer the rVSV expressing the MPXV antigens A35R, A29L, M1R, and B6R, we used the full-length VSV genome as a backbone (Fig. 1a), in which the MPXV antigens were inserted into the region between the *M* and *G* genes. The mCherry protein was fused to the N-terminus of the P protein to visualize the process of virus rescue. Surprisingly, despite attempting various transfection methods such as liposomes and calcium phosphate, we only achieved successful virus rescue using a suspension transfection (Fig. 1b and Fig. S1a). rVSV-A29L and rVSV-B6R exhibited strong fluorescence signals (F0) 72 h post-transfection. Subsequently, the supernatant was collected and subjected to blind passage (F1, F2, and F3 generations), in which similarly intense fluorescence signals were observed. However, rVSV-A35R and rVSV-M1R exhibited fluorescence signals at 96 h post-transfection in the F0 generation, with no fluorescence observed in F1 or F2 generations. However, intense fluorescence signals became detectable in the F3 generation, suggesting a delayed rescue of these recombinant viruses. These results initially illustrated the successful rescue of all of the recombinant viruses. After three consecutive rounds of plaque selection, further validation was performed by RT-polymerase chain reaction (PCR), Sanger sequencing, and Western blot to determine whether the recombinant viruses were generated (Fig. 1c and Fig. S1b). A single-cycle growth curve analysis revealed that all recombinant viruses, each carrying MPXV antigenic genes, exhibited replication kinetics in BHK-21 cells similar to those in the control group (wild type VSV, WT-VSV) at the same MOI, viral titers demonstrated rapid elevation during early infection (12–36 hpi) and stabilized at high levels (10^7^–10^8^ plaque-forming unit [PFU]/mL) with minor fluctuations, reflecting robust replication efficiency and phenotypic consistency despite the variability in virus rescue efficiency (Fig. 1d). Interestingly, the rVSVs exhibited enhanced replication kinetics compared to WT-VSV. These findings not only confirmed the authenticity of the recombinant viruses but also demonstrated that the introduction of exogenous MPXV antigens into the VSV genome not only maintained viral replication efficiency but also extended the viral replication cycle.

### Single-antigen rVSV vaccines elicit potent neutralizing antibody responses

**Fig 2:**
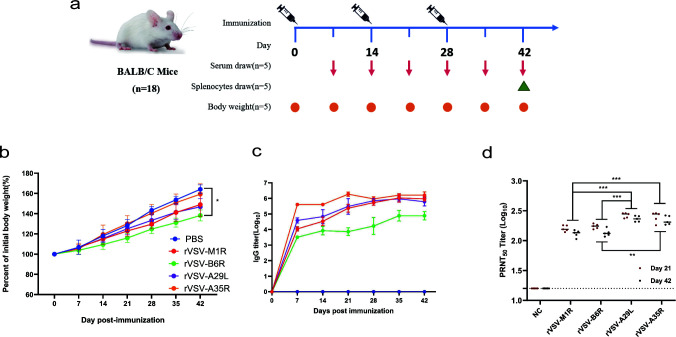
Humoral immune response levels in mice immunized with single-component rVSV vaccines. (**a**) Immunization schedule for the BALB/C mice. (**b**) Daily body weight changes of mice post-challenge. Body weight changes were calculated and compared with the initial weight. *n* = 5 biologically independent mice. (**c**) Specific IgG analysis. IgG responses in the sera on days 7, 14, 21, 28, 35, and 42 of vaccinated mice were evaluated by ELISA for binding to individually MPXV antigen proteins (A35R, A29L, B6R, and M1R). *n* = 5 per group. Threshold was set at the mean OD_450_ of negative controls + 3  SD; antigen-specific IgG remained detectable at serum dilutions up to 1: 640,000. (**d**) Neutralizing antibody analysis. Serum was tested for neutralizing antibodies against VACV using a plaque reduction neutralization test (PRNT_50_). *n* = 5 biologically independent mice. Data are presented as mean values ± SEM. Statistical significance is indicated by asterisks: ***P < 0.001 and *P < 0.05.

To eliminate potential interfering factors, the rVSVs were further purified and concentrated using sucrose density gradient centrifugation. This produced high-purity viral preparations with titers of approximately 10^9^ PFU/mL. Subsequently, the immunogenicity of the four recombinant vaccines was evaluated in mice. BALB/c mice received intramuscular injections of rVSVs at a dose of 10^8^ PFU, with booster immunizations administered on days 14 and 28 following the initial immunization (Fig. 2a). Throughout the immunization period, no significant clinical abnormalities, including ataxia, hyperexcitability, or paralysis, were observed in any of the rVSV-immunized mice. The weight of the mice remained stable throughout the study, showing a gradual increase in all groups during the immunization, consistent with the PBS control group (Fig. 2b). Mild local reactions, such as slight redness or temporary discomfort at the injection site, were noted in a few cases but resolved spontaneously within 3–5 days. Serum samples were collected on days 7, 14, 21, 28, 35, and 42 for immunogenicity analysis (Fig. 2a). To detect IgG specific to the MPXV antigens A35R, A29L, M1R, and B6R, the proteins were first expressed *in vitro* and purified, and then validated by Western blot (Fig. S2). The dynamics of A35R-, A29L-, M1R-, and B6R-specific IgG production following vaccination were determined by ELISA, as shown in Fig. 2c. Seven days after the initial immunization, specific IgG titer gradually developed in immunized mice and steadily increased over time. By day 14, the antibody response had begun to rise significantly and reached its highest levels by the seventh day following the third immunization (day 35), with a specific IgG titer ranging between 4.9 and 6.2 log10. Notably, the IgG titer induced by rVSV-B6R, initially measured at 3.5 log10, with the difference observed after day 21 (*P*<0.0001, t test), and continued to rise to 4.9 log10 by day 42, was significantly lower than IgG titer induced by other rVSVs. Among all tested vaccines, rVSV-A35R demonstrated the highest immunogenicity, inducing peak antibody titers of 6.2 log10 by day 35. These results indicated that a single-component rVSV vaccination can elicit a robust serum antibody response as early as 14 days post-inoculation, as further demonstrated by the PRNT assay results (Fig. 2d).

To assess the neutralizing activity of the vaccine, sera collected on days 21 and 42 post-primary immunization were subjected to a 50% plaque reduction neutralization test (PRNT_50_) using the homologous MPXV, VACV-WR. As expected, neutralizing activity against VACV was observed in the sera of all of the rVSV groups on day 21. The PRNT_50_ titer remained stable between day 21 and day 42, with no significant increase over time. Among the single-antigen vaccines, rVSV-A29L and rVSV-A35R induced the strongest neutralizing activity, while rVSV-M1R and rVSV-B6R generated lower titers (*P* < 0.05 for rVSV-M1R and rVSV-A29L). These differences underscore the variable immunogenicity of individual MPXV antigens in driving neutralizing antibody responses.

### Robust T-cell activation and cytokine production by rVSV immunization

**Fig 3:**
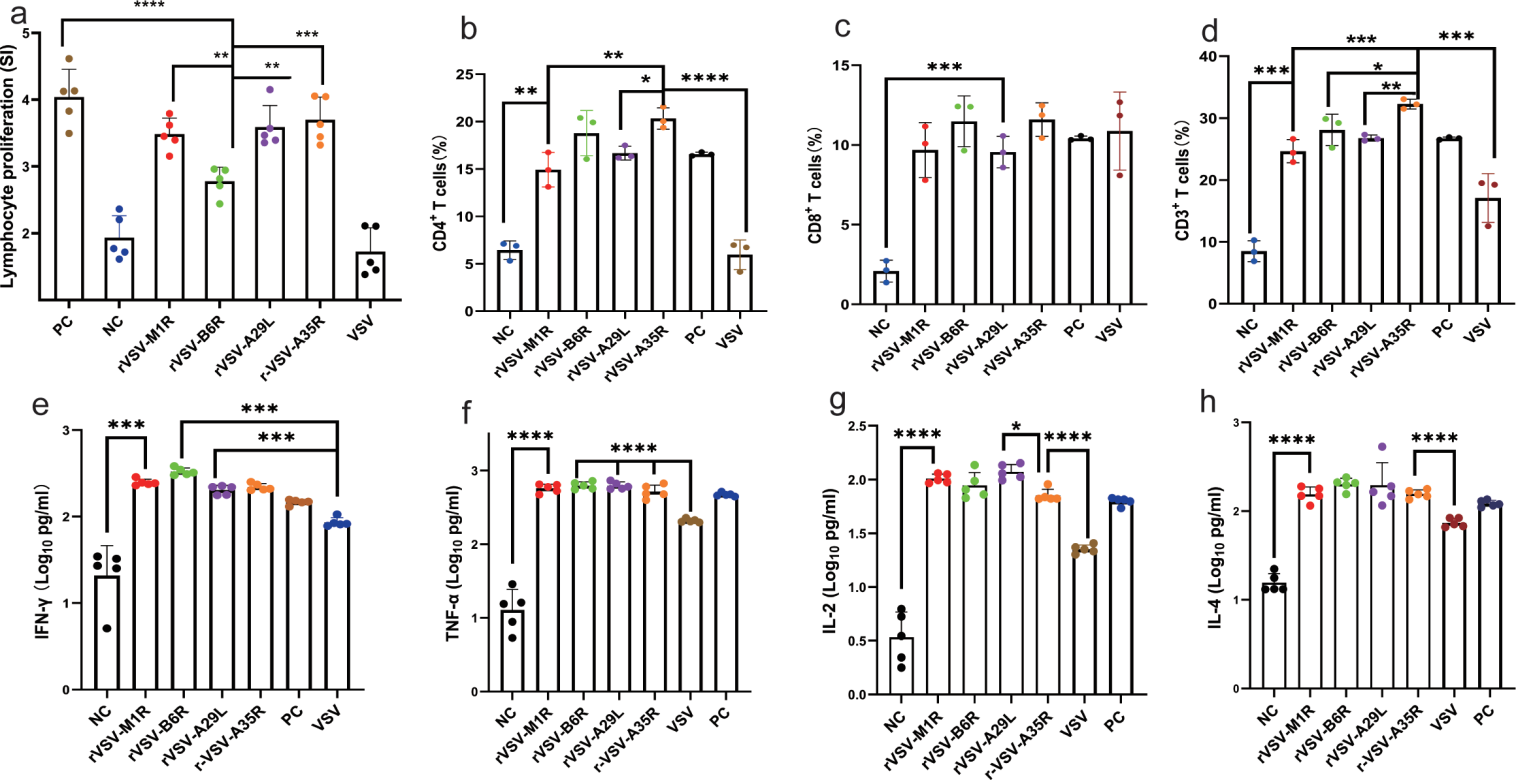
Cellular immune response levels in mice immunized with single-component rVSV vaccines. All spleen cells were isolated from mice 14 days post-triple immunization and subjected to *in vitro* stimulation with MPXV antigens (A29L, A35R, M1R, and B6R) prior to analysis. (**a**) Lymphocyte proliferation induced by single-component rVSV vaccines (*n* = 5). The positive control (PC) group stimulated mouse lymphocytes with Concanavalin A (ConA), while the negative control (NC) group stimulated lymphocytes from negative mice with the respective antigen. Each sample was tested three times. (**b–d**) Differentiation levels of specific T cells. Flow cytometry was used to assess the proportions of CD4+, CD8+, and CD3+ positive specific T cells induced by single-component rVSV vaccines in mice (*n* = 3). The data were processed and presented as percentages using the CytExpert software. (**e–h**) Cytokine detection by ELISA. Measurement of IFN-γ (**e**), TNF-α (**f**), IL-2 (**g**), and IL-4 (**h**) levels in the supernatant of stimulated lymphocytes (*n* = 5). Each sample was tested two times. Data are presented as mean ± SEM, and statistical significance was determined by Student’s *t-*test. Statistical significance is indicated by asterisks: ****P < 0.0001, ***P < 0.001, **P < 0.01, and *P < 0.05.

To investigate the cellular immunity induced by the recombinant vaccines, the spleens of vaccinated mice (*n* = 5 per group) were collected 14 days after the third booster immunization. Splenocytes were isolated from the spleens and stimulated with antigenic proteins. Initially, the proliferation of antigen-specific lymphocytes was quantitatively assessed using a CCK-8 assay. Lymphocytes from the groups immunized with rVSVs exhibited a significantly enhanced proliferative response, with a stimulation index (SI) ranging from 2.8 to 3.7, and the SI index of rVSV-B6R was also significantly lower than that of other rVSVs. The proliferation of specific types of T cells was measured by flow cytometry. The results revealed a significant increase in CD4^+^ cell proportions, rising from 6.4% in the control group to 14.9%–20.3% in the vaccine groups, representing a 2.3-fold to 3.2-fold increase (*P* < 0.0001, *t* test). Similarly, CD8^+^ cell proportions significantly increased to 9.6%–10.5%, a 4.8-fold to 5.3-fold increase compared to the 2.0% observed in the control group (*P* < 0.0001, *t* test). Additionally, the proportion of CD3^+^ cells increased significantly from 8.4% in the control group to 24.6%–32.3% in the vaccine groups, corresponding to a 2.9-fold to 3.8-fold increase (*P* < 0.0001, *t* test) (Fig. 3b through d). Notably, rVSV-A35R induces a stronger specific T-cell response compared to other rVSVs.

IFN-γ and TNF-α are critical indicators of Th1-type immune responses and provide valuable insights into the functionality of CD4^+^ Th1 cells, CD8^+^ cytotoxic T cells, and inflammatory pathway activation, all of which are integral to the modulation and execution of cell-mediated antiviral immune responses (40). ELISA analyses of the splenocytes were performed, which showed that the rVSVs elicited significant IFN-γ and TNF-α production, with levels approximately reaching up to 2.5 log_10_ pg/mL and 2.7 log_10_ pg/mL, respectively (Fig. 3e and f). Th2-type immune responses are characterized by the production of a spectrum of cytokines that drive the proliferation and differentiation of B cells, promoting antibody-mediated immunity responses. IL-2 critically maintains adaptive immune responses by regulating T-cell proliferation and immune tolerance, while IL-4 serves as the master regulator of Th2 immunity, mediating B-cell activation, anti-parasitic defense, and allergic responses to orchestrate immune homeostasis, with their dynamic interplay through synergistic and antagonistic interactions shaping the overall immunological equilibrium (41, 42). Our findings revealed that the rVSVs induced elevated levels of IL-2 and IL-4 in splenocytes, reaching concentrations of approximately 1.9 log_10_ pg/mL and 2.2 log_10_ pg/mL, respectively (Fig. 3g and h). Notably, significant differences in IL-2 production levels were observed between rVSV-A29L and rVSV-A35R (*P* < 0.05, *t* test). These results indicated that the rVSV vaccines effectively stimulated T-cell-mediated immune responses, which may contribute to establishing protective immunity against the virus.

### Multivalent rVSV cocktails synergistically enhance humoral immune response

**Fig 4:**
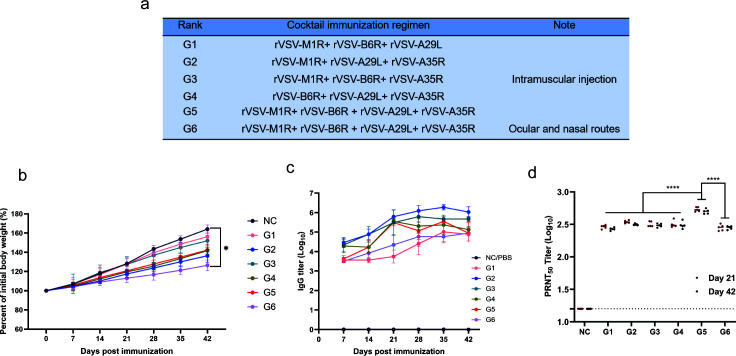
(**a**) The cocktail immunization induced high titers of IgG and neutralizing antibodies in mice. Cocktail immunization regimens and administration routes. (**b**) Weight changes in mice post-immunization, expressed as a percentage of initial body weight in experimental (G1–G6) and control (PBS) groups, with data showing weight monitoring at different time points (*n* = 5). (**c**) Serum samples from mice were collected at different times post-immunization, and IgG antibody titers specific to MPXV antigens A29L, A35R, M1R, and B6R were measured by ELISA. (**d**) PRNT_50_ titers (log_10_) in experimental (G1–G6) and control (NC) groups at 21 and 42 days post-immunization. Statistical significance denoted as **** (*P* < 0.0001), ** (*P* < 0.01), and * (*P* < 0.05) (Student’s *t*-test).

To identify vaccine groups with optimal immunogenicity, six combinations of recombinant virus cocktails were first prepared (Fig. 4a) and administered to 6- to 8-week-old female BALB/c mice (*n* = 18) at a dose of 10^8^ PFU, according to the immunization schedule shown in Fig. 2a. Similar to the single-component immunization groups, no weight loss or clinical abnormalities were observed in the cocktail immunization groups, and the body weight increased to between 126.4% and 156.2% by day 42 post-immunization (Fig. 4b). Serum antibody levels were assessed on days 7, 14, 21, 28, 35, and 42 post-immunizations (Fig. 4c). The specific IgG titer in mice in response to the cocktail vaccination exhibited a trend similar to those in response to single-component immunization, with antibody levels of most combination groups peaking at day 35 post-immunization, except for group G6. Group G2 achieved the highest IgG titer of 6.3 log_10_ on day 35, which was higher than single-component immunization groups. Additionally, sera from nearly all cocktail vaccine groups demonstrated remarkable neutralizing activity, with PRNT_50_ titers exceeding 2.4 log_10_. Notably, the G5 exhibited significantly higher neutralizing activity compared to other groups (*P* < 0.001). These results indicate that cocktail immunization induces robust humoral immune responses in BALB/c mice, while responses triggered by ocular and intranasal administration were lower than those induced by intramuscular injection (Fig. 2c and 4c).

To further compare the immunogenicity, a two-component cocktail immunization strategy was implemented via intramuscular injection (Fig. S3a). Antibody levels in response to two-component cocktails were lower than those induced by the three- and four-component cocktails, with values between 4.9 log_10_ and 5.9 log_10_ on day 35 (Fig. S3b). Notably, T6 (rVSV-A35R and rVSV-A29L) demonstrated higher IgG titers (5.9 log_10_) at day 28, while T3 (rVSV-M1R and rVSV-A35R) exhibited the highest viral neutralizing activity with PRNT_50_ of 2.7 log_10_ at day 21 (Fig. S3c).

### Cocktail vaccination enhances antigen-specific T-cell responses

**Fig 5:**
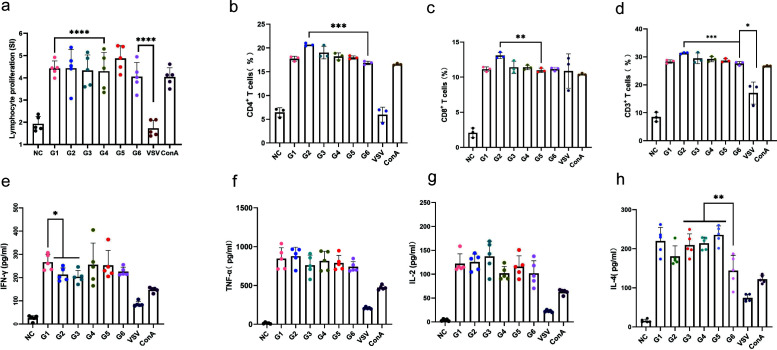
The cocktail immunization elicited robust cellular immune responses. All spleen cells were isolated from mice 14 days post-triple immunization and subjected to *in vitro* stimulation with MPXV mixed antigens (A29L, A35R, M1R, and B6R) prior to analysis. (**a**) Lymphocyte proliferation induced by cocktail vaccines (*n* = 5). Each sample was tested in triplicate. The PC group stimulated mouse lymphocytes with ConA, while the NC group stimulated lymphocytes from negative mice with the respective antigen. (**b–d**) Differentiation levels of specific T cells. Flow cytometry was used to assess the proportions of CD4^+^, CD8^+^, and CD3^+^ positive cells induced by cocktail vaccines in mice (*n* = 3). The data were processed and presented as percentages using the CytExpert software. (**e–h**) Expression levels of cytokines. IFN-γ (**e**), TNF-α (**f**), IL-2 (**g**), and IL-4 (**h**) levels in the supernatant of stimulated lymphocytes were detected by ELISA. (*n* = 5). Each sample was tested in triplicate. Data are presented as mean ± SEM, and statistical significance was determined by Student’s *t-*test. Statistical significance is indicated by asterisks: ****P < 0.0001, ***P < 0.001, **P < 0.01, and *P < 0.05.

Five mice from each group were euthanized to isolate spleen cells for further immunologic assessments. The results of a lymphocyte proliferation assay demonstrated that, compared to the PBS and rVSV single-component immunization groups, cocktail immunization induced a significantly higher level of lymphocyte proliferation, reaching 4.0–4.9, and particularly in group G5 (Fig. 5a). Next, CD4^+^, CD8^+^, and CD3^+^ T-cell proliferation were evaluated (Fig. 5b–d). As expected, cocktail immunization also induced specific T-cell proliferation. Notably, compared to single-component rVSV immunization (Fig. 3b–d), cocktail immunization induced a significantly stronger level of cell proliferation with CD4^+^ T cells at 16.8%–20.6%, CD8^+^ T cells at 10.8%–13.0% and CD3^+^ T cells at 27.6%–31.3%. Further analysis of each group revealed that group G2 induced a stronger specific T-cell response overall. The results indicated that cocktail immunization effectively promoted a stronger cellular immune response specific to MPXV antigens compared to single-component immunization. Cytokine analyses showed that the production of IFN-γ, TNF-α, IL-2, and IL-4 was stimulated in all immunized groups (Fig. 5e–h). No significant differences in the cytokine levels were observed between cocktail and single-component immunization groups. All immunized groups showed significant differences compared with the NC and the VSV empty-vector control group (*P* < 0.0001). Besides, similar to the humoral immune response, group G6 exhibited lower levels of IL-4 production compared to the other groups, with significant differences observed between G6 and G1, G3, and G5 (*P* < 0.01, *t* test). These findings suggested that cocktail immunization induced a robust cellular immune response, thereby supporting subsequent antiviral protective responses.

Similarly, the cellular immune response induced by the two-component cocktail immunization was also analyzed. The two-component cocktail also stimulated a strong specific T-cell response in BALB/c mice, with group T2 (rVSV-M1R and rVSV-A29L) inducing a higher lymphocyte proliferation SI index (Fig. S3d), and T6 eliciting a higher percentage of CD4^+^ and CD8^+^ specific T-cell production (Fig. S3e through g). Cytokine analysis revealed that all cocktail vaccines induced both Th1- and Th2-type immune responses (Fig. S3h through k). However, no consistent patterns were observed across all groups in the production levels of IFN-γ, TNF-α, IL-2, and IL-4.

### Complete protection against lethal VACV-WR challenge by rVSV cocktails

**Fig 6:**
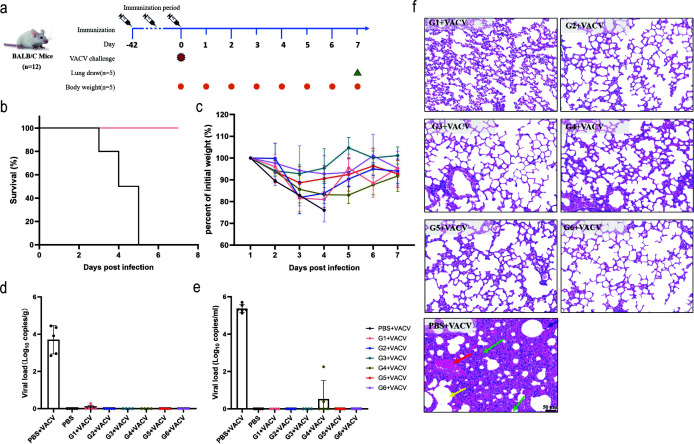
Protective efficacy of cocktail rVSV vaccines against VACV challenge in BALB/c mice. (**a**) Challenge study schedule for the BALB/c mice. (**b**) Survival curves of BALB/c mice immunized with cocktail vaccines following challenge with VACV-WR. Mice were intranasally challenged with a lethal dose (1 × 10^7^ PFU) of VACV-WR (*n* = 5). The survival status of the mice was recorded daily, and the corresponding survival rate was calculated. Death was defined as >25% wt loss. (**c**) Daily body weight of BALB/c mice immunized with cocktail vaccines following challenge with VACV-WR. Body weight changes were calculated and compared with the initial weight over 7 days post-challenge. *n* = 5 biologically independent mice. (**d and e**) Viral load of lung tissue (**d**) and serum (**e**) of BALB/c mice immunized with cocktail vaccines. On day 7 post-infection, lung tissue and serum samples were collected, and DNA was extracted. The viral load was confirmed through quantitative PCR (qPCR) analysis. *n* = 5 biologically independent mice. (**f**) Hematoxylin and eosin (HE) staining of lung sections of BALB/c mice immunized with cocktail vaccines. On day 7 post-challenge, HE staining of lung tissues from infected mice was performed. Representative histological sections from each group are shown, with 3 randomly selected fields analyzed per sample (scale bar: 50 μm). Arrows denote pathological features: lymphocytes/granulocytes in alveolar walls and spaces (green), a small number of alveoli show eosinophilic flocculent material (yellow), vascular and capillary congestion (red), and slight compensatory dilation of alveoli (blue). Data are expressed as mean ± SEM.

To assess the protective efficacy of cocktail immunization, BALB/c mice in all cocktail immunization groups, single-component immunization groups, and a PBS immunization group were intranasally challenged with 1 × 10^7^ PFU of VACV on day 42 after immunization (*n* = 12) (Fig. 6a). The weight and vital signs of the mice were continuously monitored. The results showed that the survival rate of all of the mice in the immunization group was 100%, whereas the survival rate in the PBS immunization group reduced to 0% within 3–5 days post-challenge (Fig. 6b and Fig. S4a). Immunized mice experienced a gradual weight loss during the first 4 days post-challenge but recovered thereafter, with no severe symptoms (Fig. 6c). Remarkably, single-component rVSV and two-component cocktail vaccines also achieved complete protection against the lethal VACV challenge, with similar weight recovery patterns (Fig. S4b and S5b).

On day 7 post-challenge, five mice from each group were euthanized, and the serum and lung tissue specimens were collected for subsequent analysis (43). Viral DNA was extracted from serum and lung tissue specimens, and the VACV virus load was evaluated using qPCR (Fig. 6d and e). In the PBS-immunized mice, the viral load reached 3.7 log_10_ copies/g in lung tissue and 5.3 log_10_ copies/mL in serum, indicating active VACV infection in the control group. In contrast, in the cocktail-immunized mice, almost no detectable viral genome was detected. These results demonstrated that the cocktail immunization regimen conferred robust protection against a high-level viral challenge. This was further corroborated by histopathological analysis of lung tissue using HE staining (Fig. 6f). After infection with VACV-WR, almost all of the mice in the PBS control group exhibited significant inflammatory cell infiltration in the alveolar spaces, along with alveolar epithelial cell necrosis, consolidation, fragmentation, alveolar hemorrhage, and vascular congestion. Conversely, cocktail-immunized groups showed minimal to no pathological changes. Notably, although the humoral and cellular immune responses induced in mice varied across different immunization groups, all single-component vaccine groups and two-component cocktail immunization groups exhibited nearly undetectable levels of the virus in the mice, achieving viral clearance, except for a few groups where weakly positive genomes were detected due to individual variations (Fig. S4c, d and S5c, d). Similar results were also confirmed through HE staining. Additionally, the group rVSV-M1R, rVSV-B6R, and T1 and T3 exhibited a slight increase in alveolar inflammatory cell infiltration and vascular and capillary congestion (Fig. S4e and S5e).

### Durable immunity conferred by cocktail vaccination against secondary infection

**Fig 7:**
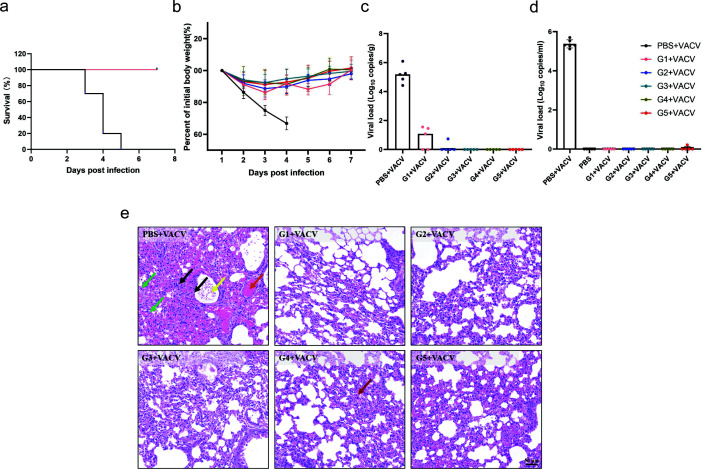
Protection of cocktail vaccination in secondary VACV challenge. Sixty days after the first VACV challenge, immunized mice were re-infected with a lethal dose (1 × 10^7^ PFU) of the VACV-WR strain. (**a**) Survival curves of BALB/c mice immunized with cocktail vaccines following secondary challenge with VACV-WR. Death was defined as >25% wt loss. (**b**) Daily body weight of BALB/c mice immunized with cocktail vaccines following secondary challenge with VACV-WR. Body weight changes were calculated and compared with the initial weight. *n* = 5 biologically independent mice. (**c and d**) Viral load of lung tissue (**c**) and serum (**d**) of BALB/c mice. On day 7 post-secondary infection, lung tissue and serum samples were collected, and DNA was extracted. The viral load was confirmed through qPCR analysis. *n* = 5 biologically independent mice. (**e**) HE staining of lung sections of BALB/c mice. On day 7 post-secondary challenge, H&E staining of lung tissues from infected mice was performed. Representative histological sections from each group are shown, with three randomly selected fields analyzed per sample (scale bar: 50 μm). Arrows indicate lymphocytes and granulocytes in the alveolar walls and alveolar lumen (green), necrosis of alveolar epithelial cells and bronchiolar epithelial cells (black), alveolar hemorrhage (yellow), vascular and capillary congestion (orange), bronchiolar hemorrhage (yellow), and alveolar macrophages in the alveolar space (red).

To further validate the protective efficacy of the cocktail immunization regimen G1–G5 against a secondary viral challenge, we performed an additional challenge test on day 60 post-primary infection. As expected, the immunized mice exhibited the same level of protection as they did during the first challenge, achieving a survival rate of 100% (Fig. 7a), with a slight decrease in body weight within the 3 dpi, and gradual recovery was observed starting from the fourth day onward (Fig. 7b). Surprisingly, viral load analysis showed that viral genomes were undetectable in the blood samples from all groups at 7 days post-secondary challenge (Fig. 7d). However, viral genomes were only undetectable in the lung tissues of the groups G2–G5, while weakly positive results were observed in three G1 samples, likely due to individual variations (Fig. 7c). A histopathological analysis yielded consistent results. In contrast, mice in the PBS control group exhibited inflammatory cell infiltration, vascular edema, and hemorrhage following virus challenge. No inflammatory responses were observed in the five cocktail-immunized groups (Fig. 7d). Overall, the cocktail immunization strategy conferred robust protection following the initial infection and elicited sustained and effective immune memory with protective efficacy upon re-exposure. These findings demonstrate the long-term durability of protection and provide crucial evidence for the safety and efficacy of cocktail vaccinations in clinical applications.

### Cocktail vaccination restricts viral genetic drift in key immune evasion genes compared to single-antigen vaccines

Poxviruses encode numerous immunomodulatory proteins to subvert host immunity. For example, the E3L protein dampens interferon-mediated antiviral signaling, thereby weakening innate immune defenses (44). Similarly, B7R, a virulence factor unique to MPXV, localizes to the endoplasmic reticulum. Although dispensable for viral replication, its deletion markedly reduces pathogenicity, suggesting a role in regulating apoptosis or interacting with immune-related host proteins (45, 46). To determine whether vaccine-induced immune pressure could drive viral escape, immunized mice underwent serial non-lethal challenges (10⁴ PFU) at 30-day intervals. The virus used for each challenge was serially passaged, originating from the lung-derived isolates of the previous challenge group. Viruses isolated 3 days after the fifth challenge were subsequently analyzed by sequencing the immune evasion-associated genes E3L and B7R. Strikingly, no mutations were detected in either E3L or B7R in the cocktail-vaccinated group (G4). In contrast, various amino acid substitutions were detected in both genes across all single-component vaccine groups (in Table 1). These results indicate that the cocktail immunization may limit the virus’s capacity for immune evasion by preventing the selection of escape mutations in key immunomodulatory proteins.

**TABLE 1:** Detection of mutations in VACV E3L and B7R genes across different immunization groups^*a*^

Group	Protein	Position (aa)	Mutation (Ref→alt)
rVSV-MIR	E3L	12; 37; 140	A→S; N→H; V→A
	B7R	4; 21; 85	K→E; N→D; I→M
rVSV-B6R	E3L	30; 42	A→P; E→A
	B7R	19; 84; 94; 143	S→T; K→R; S→T; Y→C
rVSV-A29L	E3L	73; 106; 178	D→E; *P*→T; A→T
	B7R	11; 26; 118	V→A; F→V; Y→H
rVSVA35R	E3L	47; 73; 116	L→M; D→N; A→T
	B7R	42; 60	E→G; T→P
G4	E3L	None	None
	B7R	None	None

^*a*^ Genomic DNA was extracted from mouse lung tissues on day 5 after viral challenge. Specific regions of the E3L and B7R genes were amplified by PCR and analyzed by Sanger sequencing. The obtained sequences were aligned with the reference VACV genome to identify amino acid substitutions.

## DISCUSSION

Monkeypox, a zoonotic disease, has diversified into new forms, suggesting that its complete eradication may not be feasible in the near future (47). Vaccination remains a crucial tool for preventing illness. In our study, we demonstrated for the first time the feasibility and efficacy of using a recombinant VSV-based cocktail vaccine expressing MPXV antigenic proteins to confer protection against both an initial lethal VACV-WR challenge in mice and a re-challenge. Notably, recombinant VSVs and their cocktail immunization strategies with various combinations induce a robust immune response in mice, including increased levels of specific neutralizing antibody and cellular immune markers, and the mice did not exhibit any adverse clinical symptoms during the testing. These results suggest that the rVSV-based cocktail vaccine holds promise as a candidate vaccine for MPXV.

As a highly promising vaccine vector, VSV has been approved by the FDA as a vaccine vector for the EBOV due to its established safety profile, rendering it an ideal candidate for developing other vaccines, such as human coronaviruses, Nipah virus, and Marburg virus (48–52). Here, we introduced the MPXV antigens *A35R, A29L, M1R,* and *B6R* into the VSV vector and rescued the viruses using reverse genetics. Interestingly, we encountered certain challenges during the process. The rVSVs expressing the MPXV antigens were initially difficult to rescue, requiring us to refine our methods until we successfully achieved a suspension transfection. Additionally, we observed an intriguing phenomenon: rVSV-A35R and rVSV-M1R exhibited weak fluorescence signals only in the F0 generation after 96 h of transfection, with no fluorescence observed in the F1 and F2 generations, but with the sudden appearance of strong fluorescent signals in the F3 generation (Fig. 1 and Fig. S1). The underlying cause of this rare phenomenon remains unknown at present, but it may help explain the current lack of reported recombinant MPXV vaccines. These results suggested that the insertion of the exogenous genes *A35R* and *M1R* may have affected the virus rescue efficiency in BSR cells, whereas the growth activity may have gradually recovered when rVSV was passaged in BHK-21 cells. Typically, the insertion of exogenous genes may affect the growth of WT-VSV, resulting in a certain degree of decrease in viral titer. Interestingly, in our results, the insertion of exogenous genes unexpectedly enhanced the titer of WT-VSV.

The complete replication cycle of poxviruses occurs through two distinct forms: the intracellular mature virus (IMV) and the extracellular enveloped virus (EEV). A29L and M1R are specific proteins of the IMV form, whereas A35R and B6R are specific proteins of the EEV form. These four proteins have been shown to have the potential as antigenic proteins for poxvirus in previous studies (11, 53). Furthermore, these genes encoding MPXV antigens exhibit a high degree of homology with orthologous genes from other poxviruses (>94%), such as VACV and variola virus, and offer significant cross-protection potential. Consequently, these four proteins were selected for further vaccine development and were evaluated in this study. The responses of the mice to the four recombinant vaccines showed that the vaccination regimen was well-tolerated, with no significant clinical abnormalities observed throughout the immunization period. The production of specific IgG against MPXV antigens was confirmed by ELISA, and the PRNT_50_ further validated the neutralizing activity of the sera from immunized mice (Fig. 2). The results of the humoral response analysis showed that the four rVSVs induced different levels of specific IgG *in vivo*. Among them, rVSV-*A35R* elicited the highest IgG level, while rVSV-B6R generated the lowest IgG level, suggesting marked differences in humoral immunogenicity across MPXV antigens. In addition to humoral immune responses, our study also investigated the cellular immune response induced by the recombinant vaccines. Antigen-specific lymphocyte proliferation and an increase in the percentage of specific CD8^+^, CD4^+^, and CD3^+^ T cells observed in the vaccine-immunized groups compared to the PBS group indicated that the T-cell-mediated immune response was effectively stimulated. The significant production of IFN-γ and TNF-α, critical indicators of Th1-type immune responses, further confirmed the activation of the cellular immunity. The induction of IL-2 and IL-4 by the rVSVs suggested that the Th2 immune responses were modulated, thus in part orchestrating the overall immune response. Notably, rVSV-A35R also induced a stronger cellular immune response, as evidenced by higher percentages of CD4^+^ and CD8^+^ specific T cells. Interestingly, rVSV-B6R induced weaker lymphocyte proliferation and antibody responses compared to the other constructs. This might be due to lower *in vivo* expression or stability of the B6R protein, less efficient antigen processing and presentation, or inherently weaker immunogenicity. Understanding these differences will be important for optimizing antigen selection in future vaccine design.

To optimize immune efficacy and achieve broad-spectrum protection, four rVSVs carrying different antigens were screened in various combinations, which allowed us to comprehensively compare the immunological outcomes and identify an optimal combination strategy. Our results demonstrated that immunization under the cocktail strategy induced robust IgG and neutralizing antibody responses, which were comparable to the humoral responses elicited by previously reported mRNA vaccines (54, 55). Notably, the vaccine combination groups G2 (rVSV-M1R + rVSV-A29L + rVSV-A35R) and G5 (rVSV-M1R + rVSV-B6R+ rVSV-A29L + rVSV-A35R) provided the strongest humoral immune response, of which G2 was attributed to generating sufficiently high levels of specific IgG (6.3 log_10_ on day 35), while G5 (rVSV-M1R + rVSV-B6R + rVSV-A29L + rVSV-A35R) induced the higher neutralizing activity (PRNT_50_ titer 2.7 log_10_) (Fig. 4 and Fig. S3). Additionally, three- and four-component cocktail immunizations were able to enhance the specific IgG and neutralizing antibody levels compared to those of *A35R* used as a standalone vaccine component. G5 induced higher levels of both humoral and cellular immune responses compared to the G6 group, highlighting the impact of administration routes on immune response magnitude. In addition, our cocktail immunization strategy represents the first use of viral vector vaccines for the expression of MPXV antigens. In comparison to the mRNA vaccines previously reported, the cocktail approach elicits comparable robust humoral immune responses with the same complete protection (11, 14). Although mRNA vaccines have revolutionized vaccinology, their deployment can be constrained by stringent cold-chain requirements (e.g., BNT162b2 requires –70 °C for long-term storage) (16). In this regard, the VSV-vectored platform offers a practical advantage in thermal stability, facilitating its use in settings where maintaining an ultra-cold chain is not feasible (56). This thermostability, combined with the multivalent antigen strategy, could address distribution challenges in tropical areas where MPXV remains an outbreak, making the rVSV-based cocktail a promising candidate for future development (57). To further validate this advantage, future studies could directly compare the thermal stability of rVSV-MPXV with mRNA vaccines, providing key evidence for its cost-effectiveness.

By analyzing the lymphocyte proliferation SI and the proportion of specific T cells induced by the cocktail vaccination regimen as compared to the single-component regimen, we observed that group G2 exhibited the most optimal overall cellular immune response levels (Fig. 5). Although there was not a significant difference in cytokine expression levels among the groups, the results further affirmed the efficacy of cocktail immunization for enhancing cellular immune responses to MPXV antigens. The favorable immunological outcomes of cocktail immunization, as demonstrated in our study, aligned with previous studies on the development of vaccines for the hepatitis C virus and the novel coronavirus (SARS-CoV-2) (58, 59). The underlying reason for this achievement may be because multi-antigen stimulation elicits a broad immune response. Furthermore, different antigens may function synergistically through distinct immune pathways, thereby amplifying the overall immune effect. While immune competition between different components can occur, our findings, along with those from other studies, underscore the potency of cocktail immunization for wide application in vaccine development.

The protective efficacy of cocktail immunization was then assessed in a lethal VACV challenge model. Although there were slight fluctuations in the body weight of mice within the first 3 days following the challenge, the 100% survival rate in the cocktail immunization group compared to the 0% survival rate in the PBS immunization group strongly suggested the potential of our vaccine candidate for conferring protection against MPXV infection. We compared the efficacy of the four-component cocktail vaccine in mice after administration via intramuscular injection or ocular and nasal routes. The results revealed that the ocular and nasal routes also provided 100% protection in mice (Fig. 6). The upper respiratory tract and ocular tissues are primary and susceptible sites for MPXV infection. Mucosal immunity at these portals of entry can mount a first line of defense by generating local antibodies and tissue-resident immune responses, which neutralize the virus and block its attachment to and penetration of epithelial cells. Consequently, exploring mucosal immunization strategies holds significant promise for establishing early protective barriers against MPXV infection. Notably, since a minority of monkeypox patients can be reinfected with MPXV 2 months after the initial challenge, we conducted a second challenge with a lethal dose using mice from groups G1 to G5. All mice were continuously monitored for 2 months following the primary challenge and maintained stable vital signs throughout. After the second challenge, all of the experimental groups demonstrated complete protection conferred by the cocktail vaccines. Interestingly, unlike the first challenge, the mice showed only mild weight fluctuations in the second challenge after viral infection. These results reflect the strong protective effect of the vaccines in mice, further attesting to their efficacy and durability.

To further assess whether repeated viral challenges could drive genomic alterations in immune-evasion-related proteins, DNA was extracted for sequencing after the fifth challenge. Sequencing analysis revealed a striking contrast: mutations were readily detected in all single-component groups but were completely absent in the cocktail-vaccinated group. This consistent absence of escape mutants highlights a key advantage of the cocktail strategy. We propose that the simultaneous selective pressure exerted by the cocktail vaccine on multiple immune pathways and critical epitopes broadens the immune response, preventing focused pressure on any single target. This diversification not only increases the genetic complexity required for viral escape but also raises the associated fitness costs, thereby substantially elevating the evolutionary threshold for immune escape (60). Therefore, under repeated viral exposure, the cocktail-immunized strategy effectively suppresses the emergence of escape mutations in key immunomodulatory proteins, thereby ensuring more durable and stable protective immunity. The precise molecular mechanisms underlying this broad suppression warrant further in-depth investigation.

However, our study also has certain limitations. While the use of VACV-WR as a challenge model was a necessary and well-justified surrogate due to biosafety concerns, future studies employing live MPXV challenge in non-human species would be highly informative for validating the efficacy of our vaccine candidate. A notable advantage of the cocktail vaccine lies in its broad cross-protection, which warrants further validation in subsequent studies with different strains of MPXV or other poxviruses. Furthermore, while our results are promising in a mouse model, the translation of these findings to humans remains to be demonstrated in clinical trials. Future studies with larger cohort sizes will be required to increase statistical power and to more comprehensively assess the vaccine’s safety and immunogenicity.

In summary, our study provides crucial evidence for the safety and high efficacy of recombinant VSV-based cocktail vaccinations expressing MPXV antigens, which conferred protection against VACV-WR challenge in a mouse model. Additionally, the candidate vaccine achieved antibody titers by day 14 post-immunization comparable to those induced by mRNA vaccines at day 28 (14), suggesting the potential for adopting a two-dose regimen. This highlights the need for future studies to evaluate whether conducting the challenge at day 21 could further enhance protective outcomes. Furthermore, its peak IgG titers at day 35 post-primary immunization matched the highest reported efficacy levels in current monkeypox vaccine research (43). While direct comparisons are limited by inter-study variability in experimental conditions (e.g., animal models, antigen dosing), the potent immune responses and complete protection in lethal challenge models underscore the vaccine’s potential to combat monkeypox. Our findings position the rVSV-based cocktail vaccine as a translatable candidate for clinical development, particularly in regions where cold-chain logistics limit mRNA vaccine deployment.

## MATERIALS AND METHODS

### Cell lines, viruses, and antibodies

BSR-T7 cells were propagated in DMEM (Gibco) supplemented with 5% fetal bovine serum (FBS), 1% penicillin-streptomycin, and 1 mg/mL Geneticin G418. BHK-21 and HeLa cells (American Type Culture Collection) were maintained in DMEM containing 10% FBS and antibiotics. The recombinant VACV vTF7-3 (expressing T7 RNA polymerase) was amplified in CV-1 cells using RPMI 1640 medium with 10% FBS, following established methods (61). Commercial antibodies targeting MPXV antigens (A35:40886-M0026; A29: 40891-M0036; M1R: 40904-T62; B6R: 40902-R007) were obtained from Sino Biological.

### Plasmid construction and directed mutagenesis

The plasmids pVSV, pN, pP, and pL were generously provided by Prof. Xianzhi Xia (Military Veterinary Research Institute, Academy of Military Medical Sciences). To generate the visible recombinant VSV (Indiana serotype), the mCherry open reading frame was inserted upstream of the P gene (N-terminus) via overlap extension PCR (61), creating a fusion construct designated VSV-mCherry. MPXV antigenic gene sequences (*A35R*, *A29L*, *M1R*, *B6R*) were codon-optimized and synthesized by Sangon Biotech (Shanghai), then cloned into the intergenic region between the *M* and *G* genes of the VSV genome (62). A unique *Mlu I* restriction site within this region was introduced using the QuickChange II XL Site-Directed Mutagenesis Kit (Agilent) following the manufacturer’s instructions.

### Generation of recombinant viruses expressing antigenic proteins of MPXV

BSR-T7 cells were trypsinized, infected with vTF7-3 (MOI = 5), and transfected with a plasmid mixture (4 µg full-length VSV genome, 6 µg pN, 4 µg pP, 2 µg pL, and 0.5 µg pG) using Lipofectamine 2000 (Thermo Fisher) (61). Transfected cells were plated onto 60 mm tissue culture dishes and maintained at 31°C (5% CO₂), followed by a 2-h heat shock at 41°C at 24 h post-transfection. Culture supernatants were harvested 72–96 h post-transfection, clarified by 0.22 µm filtration (Millipore), and stored at −80°C. Infectious viral titers (PFU/mL) were quantified via plaque assay on BHK-21 monolayers as established (63).

### Purification of viruses

#### vTF7.3 purification

CV-1 cells at 95% confluency were infected with vTF7-3 at an MOI of 1. After complete cytopathic effect (CPE) development, infected cells were harvested and lysed in 10 mM Tris-HCl buffer (pH 8.8). The crude lysate was purified through discontinuous sucrose gradient ultracentrifugation (36,000 × *g*, 1.5 h, 4°C). Pelleted virions were resuspended in 10 mM Tris-HCl (pH 8.8) and stored at −80°C for further studies.

#### Fluorescence-based plaque purification of rescued recombinant viruses

BHK-21 monolayers in 12-well plates were grown to 90% confluence and exposed to 10-fold serial dilutions of rescued virus diluted in serum-free DMEM. Post-adsorption (37°C, 1 h), the inoculum was aspirated, and cells were washed thrice with PBS (pH 7.4) before adding an overlay containing 0.8% low-melting agarose in DMEM. Plates were incubated for 24 h (37°C, 5% CO₂), after which three individual plaques per dilution were excised from independent wells. Agarose plugs containing isolated virions were transferred to HeLa cell monolayers in 60-mm dishes. mCherry fluorescence was monitored at 24 h post-infection using an inverted fluorescence microscope (Nikon Eclipse Ti).

#### Purification of recovered viruses

Recombinant virion purification followed literature protocols (64). To suppress defective particle generation, HeLa cells were serially passaged under low-multiplicity infection (MOI = 0.01). Virus was harvested at 24 hpi and subjected to plaque-based titration (61). After dual-speed clarification (12,000 × *g*, 5 min) to remove aggregates, concentrated virions were ultracentrifuged (30,000 × *g*, 120 min) and fractionated through a 10 mL potassium tartrate-glycerol step gradient (0%–50% tartrate, 30–0% glycerol). Target bands were collected, diluted in PBS, repleted (30,000 × *g*, 120 min), and stored at 4°C in PBS suspension.

### RT-PCR

Viral RNA isolation from infected BHK-21 cells was performed using the RNeasy Mini Kit (Qiagen, DP419) following the manufacturer’s instructions. Total RNA was reverse transcribed with random nonamer primers (Sigma) as previously established (61, 65). The 20 μL cDNA synthesis system contained the following: 10 μL RNA template, 4 μL 5× first-strand buffer, 1 μL random nonamers (Sigma), 1 μL 10 mM dNTP mix, 2 μL 0.1 M DTT, 1 μL RNaseOUT (Thermo Fisher), and 1 μL SuperScript II reverse transcriptase (Invitrogen). The reaction was carried out at 42°C for 60 min followed by enzyme inactivation at 70°C for 15 min. Target amplification was employed using Phusion high-fidelity DNA polymerase (Thermo Fisher) under standard cycling conditions.

### Western blotting

Protein immunoblot analysis followed previously established methodologies (66). BHK-21 monolayers were washed twice with cold PBS (4°C) and lysed in ice-cold RIPA buffer (Solarbio, Beijing, China) supplemented with protease inhibitors for 30 min. Cellular proteins (20 μg/lane) were separated on 12% Bis-Tris gels and transferred onto PVDF membranes (Millipore, USA). Membranes were blocked in 5% skim milk-PBST (0.1% Tween-20) for 60 min and probed with primary antibodies (mouse IgG): anti-A29L (1:1,000), anti-A35R (1:1,500), anti-M1R (1:2,000), and anti-B6 (1:2,500) (Sino Biological). HRP-conjugated goat anti-mouse IgG (1:10,000, Abcam ab6789) was used as the secondary antibody with 1-h incubation at room temperature. Following three 5-min TBST washes, immunoreactive bands were visualized using ECL substrate (Bio-Rad #1705062) under chemidoc imaging.

### Single-cycle growth curves

BHK-21 cells were seeded in 12-well culture plates (2 × 10^5^ cells/well) and maintained at 37°C until reaching 90% confluency. Cell monolayers were inoculated with recombinant viruses at an MOI of 0.01 for 60-min adsorption. After PBS washing and replacement with DMEM supplemented with 1% FBS, culture supernatants were harvested at defined intervals (0, 12, 24, 36, 48, 60, and 72 hpi) and cryopreserved at −80°C. Viral replication kinetics were analyzed by plaque assays using standardized protocols.

### Mouse cocktail immunization

To investigate the effects of cocktail immunization, mice were challenged with a lethal dose of VACV-WR 2 weeks after booster immunization. First, 4 groups of rVSVs and 11 groups of cocktail vaccines were prepared. Female BALB/c mice, aged 6–8 weeks (*n* = 18), were intramuscularly injected with 1 × 10^8^ PFU of the virus cocktail. Booster immunizations were administered at days 14 and 28 following the primary immunization. Control mice underwent PBS injections using the identical schedule. The health status of the mice was monitored daily post-immunization, and body weights were recorded every seven days to assess changes over time. Blood samples were collected weekly via the retro-orbital venous plexus for the quantification of neutralizing antibodies. At day 42 post-immunization, five mice from each group were euthanized, and splenocytes were isolated to assess the cell-mediated immune responses. All of the mice were maintained in a specific pathogen-free environment with *ad libitum* access to food and water.

### Expression and purification of monkeypox antigens

Target antigen sequences were codon-optimized for *E. coli* expression systems, with transmembrane domains removed prior to cloning into pET-28a (+) vectors containing dual His-tags. Constructs were transformed into *Rosetta* (DE3)-competent cells and plated on kanamycin-supplemented LB agar. Protein expression was induced at an OD_600_ 0f o.6–0.8 with 0.5 mM IPTG, followed by 4-h expression at 37°C. Bacterial pellets were sonicated in ice-cold PBS after centrifugation. Ni-NTA affinity chromatography with stepwise imidazole elution (20–500 mM) was employed for protein purification.

### ELISA for MPXV-specific antibody detection

Recombinant MPXV antigens (A29L/A35R/M1R/B6R) were immobilized on 96-well plates at 10 μg/mL (100 μL/well) in carbonate buffer (pH 9.6) overnight at 4°C. Plates were washed three times with PBST (0.05% Tween-20) and blocked with 3% BSA (200 μL/well, 4°C overnight). Serially diluted murine sera (100 μL/well) were incubated for 60 min 37°C, followed by PBST washes and 60-min exposure to HRP-anti-mouse IgG (1:10,000 in 1% BSA-PBST). TMB substrate reaction (RT, 20-min dark) was terminated with 2M H_2_SO_4_. Optical density at 450 nm was measured using TECAN Infinite 200 PRO, with endpoint titers defined as the maximal dilution yielding signals exceeding 2.1-fold negative control values.

### Assessment of neutralizing activity using a plaque reduction assay

The 50% plaque reduction neutralization assay (PRNT_50_) was performed on serum samples collected at weekly intervals and stored at −80°C. BHK-21 cells were seeded in 12-well plates at a concentration of 2 × 10^5^ cells per well and grown until they reached around 90% confluency. Serum samples were heat-inactivated at 56°C for 30 min, followed by serial 10-fold dilutions. Equal volumes of the diluted serum and VACV-WR virus (100 PFU/well, prepared in serum-free DMEM) were mixed and incubated at 37°C for 1 h to allow the formation of virus-antibody complexes.

The mixture was added to BHK-21 monolayers and incubated at 37°C for 1 h. After incubation, the supernatant was removed, and a 0.5% agarose overlay medium was applied to each well. The plates were incubated for 72 h at 37°C, fixed with 4% paraformaldehyde, and stained using 0.2% crystal violet.

The plaques were counted, and neutralizing antibody titers were determined based on the serum dilution that resulted in a 50% reduction in plaque numbers.

### Lymphocyte proliferation assay

The spleens were harvested at D56 post-immunization (*n* = 5/group) for lymphocyte isolation. Tissue dissociation was performed by mechanical disruption through 70 μm filters with lymphocyte separation medium (Solarbio P8860). Mononuclear cells were purified by density gradient centrifugation (500 × *g*, 30 min) using RPMI-1640, followed by erythrocyte lysis and PBS/RPMI washes. Cell density was adjusted to 2.5 × 10^6^ cells/mL in complete RPMI-1640 (10% FBS, 1% PS). Aliquots (2 × 10^5^ cells/well) were stimulated with 10 μg/mL MPXV antigens (A29L/A35R/M1R/B6R) or 5 μg/mL ConA (HyClone) at 37°C for 48 h. Splenocytes from PBS-immunized mice (NC) were seeded at 2 × 10⁵ cells/well and stimulated with a pool of four MPXV antigens (A29L, A35R, M1R, and B6R) at a final total concentration of 10 μg/mL. CCK-8 reagent (MCE) was supplemented 2-h pre-termination. OD_450_ readings (TECAN) were used to calculate proliferation indices (SI = ODstimulated/ODunstimulated).

### Cytokine detection

Splenocytes were isolated as described above, diluted to 2.5 × 10^6^ mL, and seeded on six-well plates. Stimulants were added to the wells at 0.25 μg/μL (4 μL/well), and the cells were incubated at 37°C with 5% CO_2_ for 72 h. The supernatants were collected, and production of the cytokines IFN-γ, TNF-α, IL-2, and IL-4 was quantified using commercial ELISA kits (Solarbio, SEKM-0007, 0034, 0031, 0002).

To establish a standard curve, cytokine standards were prepared using a twofold serial dilution. Samples (100 μL) were added to the sample wells, and antibody diluent (100 μL) was added to the control wells. Detection antibodies (50 μL) were added to each well, and the plates were incubated at 37°C for 90 min, followed by four washes. Streptavidin-HRP (100 μL) was dispensed into each well and incubated at 37°C for 90 min. Subsequently, TMB substrate (100 μL) was added and allowed to react in the dark for 5 min. The reaction was terminated by the addition of 100 μL of stop solution. The OD values at 450 nm were then recorded, and the cytokine concentrations were calculated based on the standard curve.

### Flow cytometry analysis

Isolated splenocytes (1 × 10⁶ cells/tube) were washed twice with PBS and stained with fluorochrome-conjugated antibodies: fluorochrome-conjugated antibodies: FITC-anti-CD3 (BioLegend 100204), PE-anti-CD4 (100205), and APC-anti-CD8a (100408) under light-protected conditions (4°C, 30 min). Cells were pelleted (300 × *g*, 5 min), washed twice, and resuspended in 200 μL PBS. Samples were analyzed on a Beckman Coulter CytoFLEX with acquisition thresholds set at 10⁵ events/sample. Data were analyzed using FlowJo v10.7.1 with sequential gating strategies.

### Experimental mouse challenge

On day 42 after the initial inoculation, 12 mice per group were inoculated with 1 × 10^7^ PFU of VACV-WR via both intranasal and ocular routes. Body weight, survival, and clinical signs were monitored daily, including activity level, posture, fur condition, food and water intake, and respiratory status. In accordance with animal welfare guidelines, mice exhibiting a weight loss exceeding 20% or severe symptoms were euthanized. Lungs from animals that succumbed before day 7 were collected at the humane endpoint and stored at –80°C, while the lungs from five surviving mice per group were collected on day 7 post-inoculation. All samples were subsequently processed for viral load determination and histopathological analyses. To assess long-term protection, a subset of vaccinated mice was observed for 60 days and then subjected to a rechallenge under the same conditions to evaluate vaccine durability.

### Viral load in tissue and serum

Viral DNA quantification in pulmonary specimens was performed through qPCR. Genomic DNA was isolated using the Eltbio magnetic bead-based purification system (Inlaidun Biosciences). Tissue homogenization was initiated by suspending 10 mg lung parenchyma in 500 μL lysis buffer supplemented with 20 μL proteinase K, then incubating at 58°C for 16 h. 200 μL of serum was mixed with 20 μL proteinase K. After centrifugation at 13,000 × *g* for 5 min, 400 μL of clarified lysate was mixed with 400 μL binding solution and 20 μL magnetic nanoparticles. Sequential washes were performed, and purified DNA was eluted in 100 μL eluent at 56°C and stored at −80°C.

The qPCRs were conducted in a 10 μL volume, comprising 1 μL of DNA, 0.4 μL of a probe (5′-FAM ATTTCATCCCCATGCCTTTTACATTCC 3′-BHQ1), 0.8 μL each of the forward (5′-TAAGTTTCTTTTCTAGATCGGCAAC-3′) and reverse (5′-AAAGGAGCTGGAACGATA- TTTC-3′) primers, 5 μL of 2×GUeasy Taq U+ qPCR master mix (General Biosystems), and 3 μL of RNase-free water. The amplification protocol was as follows: 95°C for 2 min, followed by 42 cycles of 95°C for 10 s, and 60°C for 30 s (FAM channel). Viral loads were calculated using a standard curve generated from a 10-fold serial dilution of plasmid standards with an initial concentration of 1 × 10^7^ copies/μL.

### Histopathology

Seven days after both the initial and second inoculations, three mice from each group were randomly selected and euthanized. Lung tissue was collected, fixed in 4% paraformaldehyde, embedded in paraffin, sectioned, and stained with HE for a comprehensive histopathological analysis.

### Successive challenges in BALB/c mice and sequence analysis of lung tissue

Mice were successively challenged with non-lethal doses after completing the immunization regimen with either single-component vaccines (rVSV-M1R, rVSV-B6R, rVSV-A29L, and rVSV-A35R) or a four-component mixed vaccine. The initial challenge was performed intranasally with 10⁴ PFU of a standard VACV stock. Subsequent challenges, conducted at 30-day intervals, utilized virus populations isolated from the lungs of mice in the previous challenge round. Specifically, within 3 days post-challenge, lung tissues were aseptically collected and homogenized. The homogenates were centrifuged, and the supernatants were inoculated onto monolayers of CV-1 cells for virus isolation and amplification. After incubation for 48–72 h until a significant CPE was observed, the virus was harvested by freeze-thawing the cell culture and followed by two additional passages for expansion. The viral titer (PFU/mL) of the lysate was determined by plaque assay on HeLa cells. This stock was then used as the inoculum for the next round of challenge, with the dose normalized to 10⁴ PFU per mouse.

Genomic DNA was extracted using the TIANGEN DNA extraction kit (TIANGEN, China) according to the manufacturer’s instructions. DNA concentration and purity were measured, and the DNA was used as the template for PCR amplification with gene-specific primers (see Table S1). The resulting PCR products were separated by agarose gel electrophoresis, and bands of the expected size were excised and purified for sequencing by Sangon Biotech (Xi’an, China). Sequence alignments and variant analysis were performed to identify mutations. Mutations observed in more than two mice within the same group were recorded and summarized.

### Statistical analysis

Data analysis was performed using GraphPad Prism (version 10, GraphPad Software) and SPSS (version 24.0, Software SPSS Inc.). Data in graphs are presented as the mean ± SEM. Group comparisons were conducted using two-tailed unpaired *t*-tests for single-factor analysis or two-way ANOVA for dual-factor analysis. A significance threshold of *P* ≤ 0.05 was applied, with specific *P* values detailed in figure captions. Each experiment was repeated a minimum of three times independently.

## Data Availability

The data sets generated during and/or analyzed during the current study are available from the corresponding author on reasonable request. The data will be publicly released upon acceptance of the manuscript for publication.

## SUPPLEMENTAL MATERIAL

Supplemental material mbio.03200-25-s0001.docx Fig. S1 to S5; Table 1.

ASM does not own the copyrights to Supplemental Material that may be linked to, or accessed through, an article. The authors have granted ASM a non-exclusive, world-wide license to publish the Supplemental Material files. Please contact the corresponding author directly for reuse.
