# Supplementary material for: A cocktail vaccine with monkeypox virus antigens confers protection without selecting mutations in potential immune evasion genes in the vaccinia WR strain challenge
Source: mBio. 2025 Dec 31;17(2):e03200-25. doi: 10.1128/mbio.03200-25 (PMC12892989; doi:10.1128/mbio.03200-25)
Supplement: Supplemental material — Fig. S1 to S5; Table 1. [file mbio.03200-25-s0001.docx]

**A cocktail vaccine with monkeypox virus antigens confers protection without selecting mutations in potential immune evasion genes in vaccinia WR strain challenge**

Xintong Sun^1,2†^, Luhua Zhang^1,2†^, Guohua Chen^1,2^†, Fan Yang^3†^, Xiaoyu Ning^1,2^, Jinxin Qiu^1,2^, Yuxuan Gao^4^, Jianshe Yang^1,2^, Wenhui Zhang^1,2^, Zihui Zhang^5^, Yueyue Zhang^1,2^, Siyuan Li^1,2^, Mingcong Zeng^6^, Baoquan Fu^1,2*^, Yongfeng Li^4*^, Chen Peng^5*^, Weike Li^1,2*^

*Corresponding author.: Baoquan Fu^1,2*^, Yongfeng Li^4*^, Chen Peng^5*^, Weike Li^1,2*^

Email: fubaoquan@caas.cn (B.F.), liyongfeng@caas.cn (Y.L.), pengchenea@cau.edu.cn (C.P.), liweike@caas.cn (W.L.)


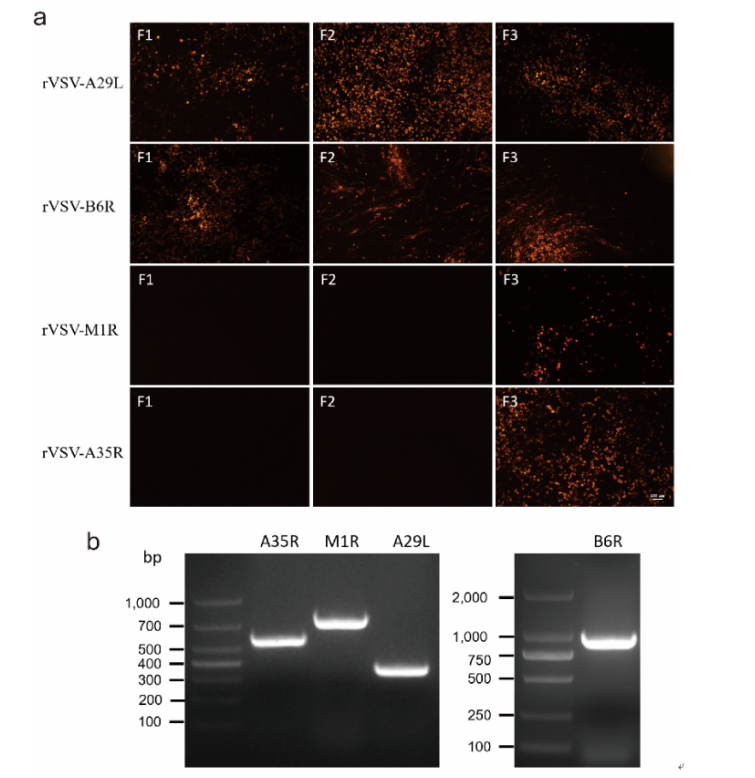


Fig. S1. Characterization of recombinant VSV (rVSV) passages and MPXV antigen expression.

a. Passage validation of rVSVs. BHK-21 cells were infected with supernatant from initially rescued virus culture, generating F1 to F3 virus passages. Five fields of view were analyzed for each virus.

b. Confirm of MPXV antigens gene by RT-PCR. BHK-21 cells were infected with rVSVs at an MOI of 1.0. At 24 h post infection, the total RNA of cells was extracted using the Trizol method. After reverse transcription, validation was performed using RT-PCR, and results were visualized through nucleic acid gel electrophoresis, the sizes of each gene were A35R (546 bp), A29L (397 bp), B6R (986 bp), and M1R (753 bp).


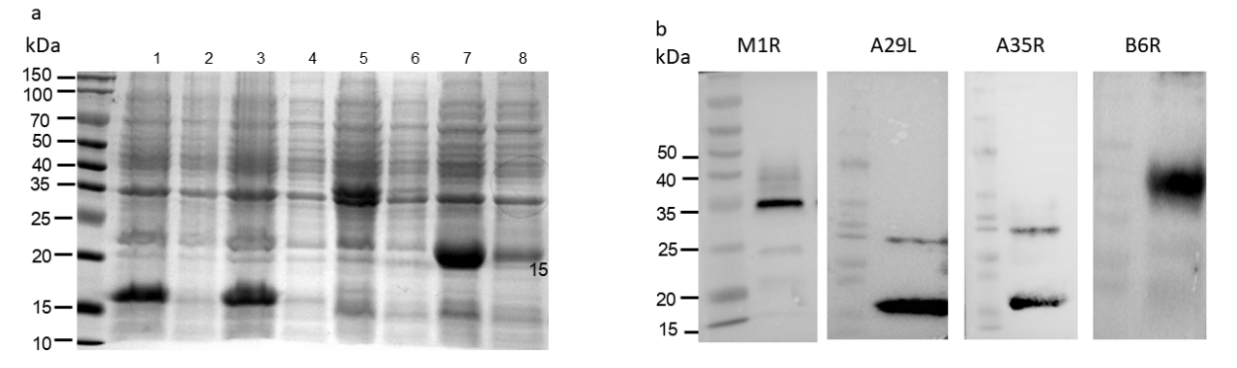


、

Fig. S2. Recombinant proteins were efficiently and specifically expressed in E. coli Rosetta.

a. SDS-PAGE analysis of recombinant protein expression in E. coli Rosetta cells. Lanes 1, 3, 5, and 7 show IPTG-induced expression of A35R (20.0 kDa), A29L (14.5 kDa), B6R (36.2 kDa), and M1R (27.6 kDa), respectively. Lanes 2, 4, 6, and 8 show corresponding uninduced controls. Molecular weight markers (kDa) are indicated on the left. Arrowheads indicate bands corresponding to the expected molecular weights of each recombinant protein.

b. Western blot analysis confirming specific expression of recombinant proteins (A35R, A29L, B6R, M1R) in IPTG-induced *E. coli* Rosetta cells.


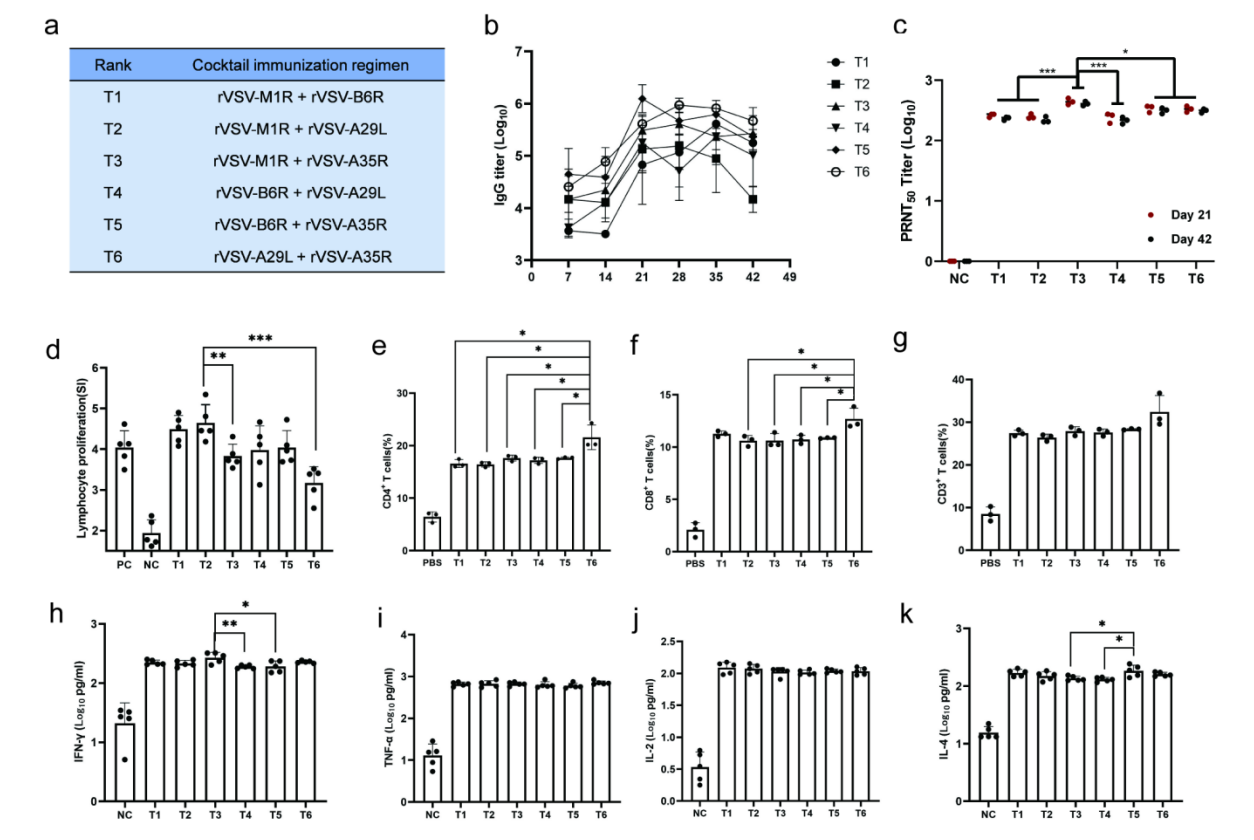


Fig. S3. The two-component rVSV cocktail vaccine elicited robust humoral and cellular immune response in mice.

a. Immunization regimens and administration routes for two-component rVSV vaccines.

b. Serum samples from mice were collected at different days post-immunization, and IgG antibody titers specific to MPXV antigens A29L, A35R, M1R, and B6R were measured by ELISA (n=5).

c. PRNT_50_ titers (Log_10_) in experimental (T1-T6) and negative control (NC) groups at 21- and 42-days post-immunization (n=3).

d. Lymphocyte proliferation induced by two-component cocktail vaccines (n=5). Each sample was tested in triplicate. The positive control (PC) group was stimulated with ConA, while the negative control (NC) group stimulated negative mice with the respective antigen.

e.f.g. Differentiation levels of specific T cells. Flow cytometry was used to assess the proportions of CD4^+^, CD8^+^, and CD3^+^ positive cells induced by two-component cocktail vaccines in mice (n=3). The data were processed and presented as percentages using the CytExpert software.

h.i.j.k. Cytokine expression levels. IFN-γ (h), TNF-α (i), IL-2 (j), and IL-4 (k) levels in the supernatant of stimulated lymphocytes (n=5) were quantified by ELISA. Each sample was tested in triplicate.

Data are presented as mean ± SEM, and statistical significance was determined by Student’s t-test. Statistical significance is indicated by asterisks: **** p<0.0001, *** p<0.001, ** p<0.01, * p<0.05.


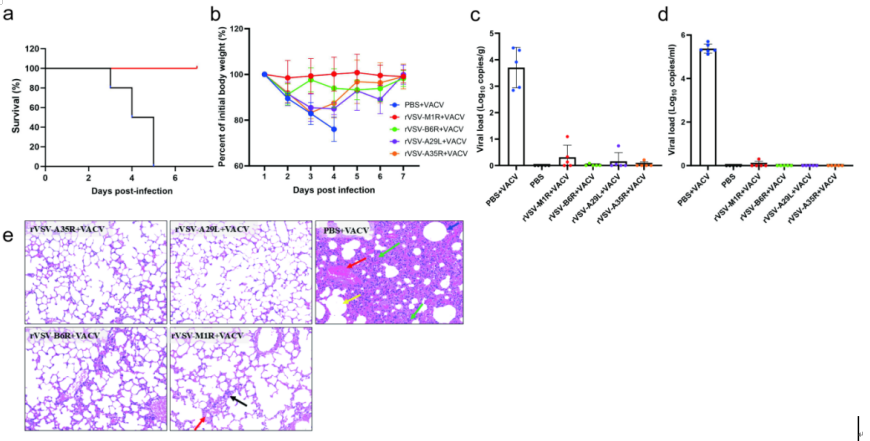


Fig. S4 Protective Efficacy of Single-Component rVSV Vaccines Post VACV Challenge.

1. Survival curves of BALB/c mice immunized with single-component cocktail vaccines following challenge with VACV-WR. Survival status was monitored daily, and survival rates were calculated. Death was defined as >25% weight loss.
2. Daily body weight of BALB/c mice immunized with single-component cocktail vaccines following challenge with VACV-WR. Body weight was measured daily and compared with the initial weight. n=5 biologically independent mice.

c.d. Viral load quantification in lung tissue (c) and serum (d) of BALB/c mice immunized with single-component rVSV vaccines. On day 7 post-infection, lung tissue and serum samples were collected and DNA was extracted for qPCR analysis. n=5 biologically independent mice.

e. Histopathological analysis of lung sections from BALB/c mice immunized with single-component rVSV vaccines. On day 7 post-challenge, lung tissues were collected and stained with hematoxylin and eosin (H&E). Representative images from each group are shown, with 3 randomly selected fields analyzed per sample (scale bar: 50 μm). Arrows indicate lymphocytes and granulocytes in the alveolar walls and lumen (black), and vascular/capillary congestion (red).

Data are expressed as mean ± SEM.


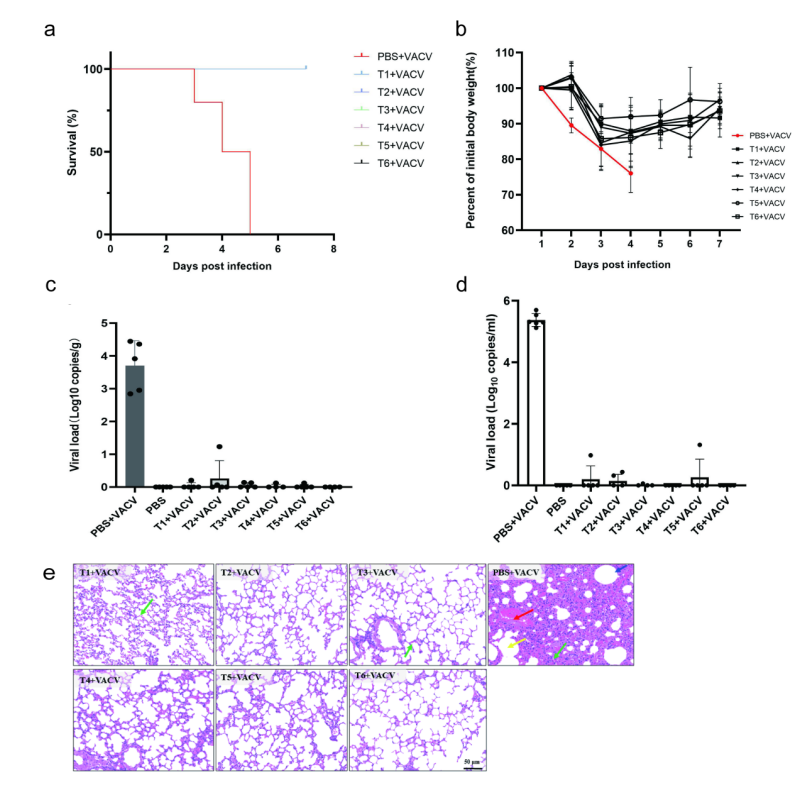


Fig. S5 Protective Efficacy of two-component Vaccines Post-VACV Challenge.

a. Survival curves of BALB/c mice. Mice were intranasally challenged with a lethal dose (1×10^7^ PFU) of VACV-WR. Survival status was monitored daily, and the corresponding survival rates were calculated. Death was defined as >25% weight loss.

b. Daily body weight changes in BALB/c mice. Body weight was measured daily and compared with the initial weight. n=5 biologically independent mice.

c.d. Viral load quantification lung tissue (c) and serum (d) of BALB/c mice. On day 7 post-secondary infection, lung tissue and serum samples were collected, and DNA was extracted for qPCR analysis. Data represent n=5 biologically independent mice.

e. Histopathological analysis of lung sections from BALB/c mice. On day 7 post-challenge, lung tissues were collected and stained with hematoxylin and eosin (H&E). Representative images from each group are shown, with 3 randomly selected fields analyzed per sample (scale bar: 50 μm). Arrows indicate lymphocytes and granulocytes in the alveolar walls and alveolar lumen (green), necrosis of alveolar epithelial cells and bronchiolar epithelial cells (black), alveolar hemorrhage (yellow), vascular and capillary congestion (orange), bronchiolar hemorrhage (yellow), and alveolar macrophages in the alveolar space (red).

**Supplementary Table 1**

Primers used for amplification of the E3L and B7R genes, with sequences listed in the 5′ to 3′ orientation.

| Primer name | Sequence（5′→3′） |
| --- | --- |
| B7R-F | CTAATGTATGATATAATTAATAGCGTATC |
| B7R-R | CCTTCTGGTACATTTGTATACATTG |
| E3L-F | GGGTAGATAAGTGGGATGAAAT |
| E3R-R | GAGCATTTCGCAATCTTAATG |
